# Cognitive-Emotional and Inhibitory Deficits as a Window to Moral Decision-Making Difficulties Related to Exposure to Violence

**DOI:** 10.3389/fpsyg.2019.01427

**Published:** 2019-07-17

**Authors:** Micaela Maria Zucchelli, Giuseppe Ugazio

**Affiliations:** ^1^Department of Psychology, University of Bologna, Bologna, Italy; ^2^Moral Psychology Research Lab, Department of Psychology, Harvard University, Cambridge, MA, United States; ^3^Geneva Finance Research Institute, University of Geneva, Geneva, Switzerland

**Keywords:** moral decision-making, empathy, theory of mind, inhibitory control abilities, mental health

## Abstract

In the present review, we illustrate how exposure to violence results in an increased probability of developing functional impairments of decision mechanisms necessary for moral behavior. We focus in particular on the detrimental effects of exposure to violence on emotional (e.g., Empathy), cognitive (e.g., Theory of Mind), and inhibitory control abilities. Relying on studies that document impaired moral behavior in individuals with deficits in these abilities, we propose a “model” of how exposure to violence can affect moral behavior. We then discuss how impaired moral decision making can also be a factor increasing the likelihood of reiterating violence: agents who lack abilities such as understanding and resonating with others’ emotions or inhibitory control, can lead to an increase of violent displays. Thus, if not properly addressed, the noxious effects of exposure to violence on morality can lead to a violence generating cycle. We conclude proposing that interventions targeted at improving moral behavior can maximize their efficacy focusing on mitigating the impact of violence on the basic cognitive, emotional, and inhibitory abilities discussed.

## Introduction

Human societies have been characterized by persistent exposure to violence and warfare (Krug et al., 2002). While some scholars propose that violence has been steadily diminishing throughout human history (Pinker, 2011), in the last century almost 200 million people have lost their lives directly or indirectly as a result of violent conflict, and a larger number has been exposed to violence over a long period of time, developing physical and/or mental health problems (World Health Organization, 2002). In particular, it has been estimated that up to 1 billion children (aged 2–17 years) have been victims of emotional, physical, or sexual violence in just 1 year (Hillis et al., 2016). Violence exposure can, indeed, happen in multiple settings, such as within private relationships (e.g., within families, friends, peers, etc.) or communities (e.g., at school, neighborhoods, etc.) (World Health Organization, 2002).

Importantly, exposure to violence not only has immediate negative consequences on people’s physical and mental health, but it can also have longer-term impacts on human’s decision-making abilities. These long-term effects are more likely to be manifest in younger individuals, as they can negatively impact the development of mental functions. For instance, traumatic childhood experiences of violence have been shown to result in increase in the likelihood of developing personality disorders such as antisocial, borderline, or schizotypal during adolescence or early adulthood (Johnson et al., 1999; Battle et al., 2004). Crucially, during this delicate time in life the effects of violence on mental health are most worrisome since children are developing their initial cognitive, emotional and social behavioral skills through their first relationships with parents and peers (Osofky, 1999). In particular, they are developing their moral reasoning, i.e., the process through which a person determines what is right (and what is wrong) in specific situations given the norms accepted in society. Therefore, children learn to act in accordance with the accepted standards, for instance without harming others (e.g., Kohlberg, 1958).

Several scholars have documented the negative effects of violence exposure on children’s moral development (e.g., Leavitt and Fox, 1993; Cairns and Dawes, 1996; Garbarino and Kostelny, 1996; Macksoud and Aber, 1996; Posada and Wainryb, 2008; Ardila-Rey et al., 2009). For instance Ardila-Rey et al. (2009) found that children exposed to extreme violence were more likely to condone moral transgressions (such as hitting others or not sharing toys) when provoked or for reasons of retaliation, compared to children who had low exposure to violence, even if both groups condemned an unprovoked moral transgression. Likewise, Posada and Wainryb (2008) indicated that children and adolescents, who have been exposed to some form of community violence, judged morally acceptable both stealing and physically harming others in contexts of survival or revenge. Finally, Posada (2012) showed that children who were exposed to violent events, judged in a positive way the act of causing harm to someone else in a context of vengeance, considering such act as a way to restore justice.

Despite the evidence from these initial studies, the literature is still lacking a sufficiently rich body of studies characterizing the underlying mechanisms that explain how violence affects moral reasoning and behavior. Therefore, in this paper we examine how violence impairs the ability to make moral decisions determining deficits in three of the mechanisms necessary for moral behavior (Buon et al., 2016): emotions (Haidt, 2001, 2007; Reniers et al., 2012; Ugazio et al., 2012, 2014), cognition (Young et al., 2007) and inhibitory control abilities (Greene et al., 2008; Moore et al., 2008). The importance of these mechanisms for moral decision making is underscored by a rich body of studies: in line with Haidt’s *Moral Intuitionist Model*, scholars have analyzed how basic emotions, such as anger and disgust among others (Haidt, 2001; Wheatley and Haidt, 2005; Prinz, 2006; Schnall et al., 2008), and more complex emotions, such as empathy (Pizarro, 2000; Reniers et al., 2012; Buon et al., 2016) can influence our moral judgments. On the other hand, studies supporting the *Moral Rationalist Model* (Piaget, 1965; Kohlberg, 1969; Turiel, 1983) have discussed how cognitive mechanisms, such as those necessary to estimate the intentionality of agents or value estimations about the outcomes of a certain action (e.g., Baird and Astington, 2004; Knobe, 2005; Borg et al., 2006; Cushman et al., 2006; Mikhail, 2007; Young et al., 2007; Shenhav and Greene, 2010; Trémolière and Bonnefon, 2014) play a crucial role in moral decision making. Importantly, to date, the consensus in moral neuro-cognitive psychology reconciles these contradictory positions, proposing that human morality is a construct consisting of multiple complex processes comprising emotions, cognitive processes and executive control abilities (Greene et al., 2004, 2008; Cushman et al., 2007; Cushman, 2008; Greene, 2009; Bzdok et al., 2012; Buon et al., 2016). Thus, selecting the morally appropriate behavioral response to a situation requires to be sensitive to emotional inputs, understanding and integrating information about goals and intentions and inhibiting unnecessary or confounding elements in the specific situation (such as emotional reaction in the case of accidental harms) (Buon et al., 2016). Disruption of any of these systems, and hence of their functional interactivity, results in impaired moral decision making (e.g., Mendez et al., 2005; Koenigs et al., 2007; Young et al., 2010; Ciaramelli et al., 2012).

In this paper, we propose a more comprehensive overview of how violence can impact moral decisions adopting an indirect route: we first illustrate how exposure to violence leads to impairment in three different types of decision mechanisms necessary for morality, i.e., emotional, cognitive, and inhibitory control (Buon et al., 2016). We then consider different personality conditions, each associated with impairments in one of those abilities, as a “model” for understanding which consequences arise in the moral reasoning and behavior of people with atypical affective, cognitive and inhibitory processing (Kooiman et al., 2004; Rogosch and Cicchetti, 2005; Lewis et al., 2015; Dargis and Koenigs, 2017; Kersten et al., 2017; Vallejos et al., 2017). Through this strategy we can therefore characterize how moral behavior can be impaired by functional deficits in emotional, cognitive and inhibitory control mechanisms resulting from exposure to violence. Specifically, we propose that individuals who suffer deficits in empathy due to exposure to violence (Guo et al., 2013) are likely to display moral behaviors observed in individuals with *psychopathic* and *alexithymic personality* traits, as these traits have been primarily associated with diminished empathy ability (Blair et al., 2001; Blair, 2006; Moriguchi et al., 2007; Bird et al., 2010); in a similar vein, individuals with *Borderline* and *Schizophrenic personality disorders*, characterized by deficits in perspective-taking ability (Sprong et al., 2007; Sharp et al., 2011), are proposed to reflect the expected moral behavior of victims of violence who develop deficits in Theory of Mind ability (Nazarov et al., 2014). Thirdly, individuals who see their inhibitory control abilities impaired due to exposure to violence (Bogliacino et al., 2017), are proposed to display moral behaviors resembling those of individuals diagnosed with *Attention Deficit/Hyperactivity disorder (ADHD)* and *Antisocial Personality disorder*, both characterized by reduced inhibitory abilities (Morgan and Lilienfeld, 2000; Willcutt et al., 2005). Note that along the manuscript *disorders* or *traits of personality* will be introduced: each time it will be specified which of them we are referring to, according to the studies discussed.

Importantly, we also stress that individuals with impaired moral reasoning are also more prone to engage in hostile behaviors, likely due to their deficits in the above-mentioned mechanisms, thus increasing the presence of violence exposure within the social environments (Spenser et al., 2015). Thus, if not properly addressed, the noxious effects of exposure to violence on morality can lead to a violence generating cycle affecting, not only a person’s health, but being also detrimental for societies as they entail huge costs for the countries’ economies, employing billions in health care and law enforcement (World Health Organization, 2002). Based on the studies discussed in the present review, we suggest that interventions targeted at improving moral behavior in victims of violence can maximize their efficacy focusing on mitigating the impact of violence on the basic cognitive, emotional, and inhibitory abilities discussed.

## Empathy and Moral Decision-Making

### Exposure to Violence and Deficit in Empathy

Empathy is typically defined as the process through which one’s emotions resonate with the ones of someone else (Davis, 1994; de Vignemont and Singer, 2006). This view is supported by recent works in social neuroscience revealing that the neural networks activated when a person experiences first-handedly an emotion, are also involved when such person is asked to predict the emotional states of others (cf. the *shared network hypothesis*; Decety and Sommerville, 2003; Hein and Singer, 2008; Singer and Lamm, 2009; Lamm et al., 2011).

However, according to recent cognitive and neuroscientific theoretical models, empathy is no longer considered only as a single process (Decety and Cowell, 2014). Instead, it is suggested that empathy comprises multiple and dissociable systems that interact and operate in parallel: in this context, the one characterized above corresponds to *emotional empathy*, which involves sharing emotional states; in addition to this emotional component, scholars have also defined *cognitive empathy*, which involves understanding others’ emotional states, as well as a third component, i.e., *motivational empathy*, which involves one’s motivation to care for others, for example acting to reduce another person’s suffering (Zaki and Ochsner, 2012; Morelli et al., 2014). The distinction between the affective and cognitive systems of empathy is also supported by neuroscientific evidence, showing that individuals with damage to the ventromedial prefrontal cortex (vmPFC) are specifically impaired in cognitive empathy, whereas patients with lesions in the inferior frontal gyrus (IFG), insula, amygdala, or anterior cingulate cortex are impaired in affective empathy and emotion recognition (e.g., Shamay-Tsoory et al., 2009; Shamay-Tsoory, 2010, 2015). However, as numerous studies looking at empathy and morality focused on the emotional system (e.g., Spenser et al., 2015; Buon et al., 2016), in what follows we will focus solely on the affective component of empathy, relying on the theoretical model proposed by Shamay-Tsoory et al. (2009, 2010).

A growing body of research (e.g., Carnagey et al., 2007; Fanti et al., 2009; Anderson et al., 2010; Bushman and Anderson, 2010) demonstrates that prolonged exposure to violence can disrupt the association between others’ distress and negative emotional valence of violent actions. Exposure to violence is therefore associated with an impaired development of the ability to resonate with others’ emotions. These studies proposed that such lack of association results through a mechanism of *desensitization*. *Desensitization* is defined as a decreased cognitive, emotional, or behavioral response to a stimulus after repeated exposure to it (Rule and Ferguson, 1986). For instance, in the case of stress, the first experience of a stressful stimulus elicits a response in the individual, whereas following a repeated exposure to such stressor occurs a numbing of emotional reactions, meaning that a person’s stress response is desensitized (Gaylord-Harden et al., 2017). This mechanism is being commonly leveraged by behavioral therapies used to reduce emotional responses to phobic and aversive stimuli through graded exposure to anxiety-inducing stimuli (Wolpe, 1973). According to the pathologic adaptation model (PAM; Ng-Mak et al., 2002), the same occurs when individuals are exposed from childhood to high levels of violence: after an initial increased emotional reaction, some develop emotional numbing to violence as a coping strategy (Ng-Mak et al., 2002). By reducing emotional reaction to distress, emotional desensitization in turn disrupts the ability to empathize with other’s pain and suffering (Guo et al., 2013).

Crucially, reduced empathy for pain has been consistently found not only for long-term media violence exposure (Funk et al., 2003; Bartholow et al., 2005) but also for short-term violence exposure (Guo et al., 2013). More in detail, in the study by Guo et al. (2013), participants were exposed to either 5-min violent or non-violent video clips while their neural activity was measured with functional magnetic resonance imaging (fMRI). Behaviorally, participants who had viewed violent videos rated the pain of others to be less severe, compared to participants viewing non-violent videos. These behavioral differences were also mirrored at the neural level, as the results revealed that participants who viewed violent videos had reduced neural activations in regions encoding empathic responses to others’ pain, such as the bilateral anterior Insula (AntIns) and the anterior left midcingulate cortex (MCC). Importantly, other studies have also demonstrated a longer term (12 months) detrimental effect of exposure to violence, in the form of habitual usage of violent media genres (movies, TV series, and video games), on self-reports measuring physical and relational aggression and level of empathy (Krahé and Möller, 2010). In particular, this study found that exposure to media violence increased the likelihood of later displays of physical aggression, reducing empathy toward others.

These behavioral results have been further supported by neural data revealing that children, who have been exposed to violent environments compared to children who grew up in neutral environments, were found to have reduced thickness or gray matter volume in brain regions involved in the emotional processing of social information, including the orbitofrontal cortex (OFC, Kelly et al., 2013), the anterior cingulate cortex (ACC, Dannlowski et al., 2012) and the ventromedial Prefrontal Cortex (vmPFC) (De Brito et al., 2013; McLaughlin et al., 2014; Gold et al., 2016). In the next section the crucial role of Empathy for moral decision-making ability will be discussed.

### Empathy and Moral Decision-Making

The ability to empathize plays a very important role in the development of morality, providing a direct feedback to the observer about the emotional consequences of an action: through empathy a person can learn the emotional states of others as a result of a given action. Therefore, humans are suggested to learn which actions are considered morally good or bad largely based on the emotional consequences of a given action for the well-being of others: bad actions are associated with those that lead to distress in others, while good actions are associated to those that lead to positive emotional displays (Ugazio et al., 2014).

Indeed, Blair (1995) proposes that humans possess an *innate violence inhibition mechanism* responsible for inhibiting aggressive attacks in the presence of emotional cues signaling distress. This mechanism is evident among 4 to 7-year-old children, where display of distress in a victim (i.e., a sad facial expression) generally results in termination of aggression (Camras, 1977). Importantly, the aversion to actively causing distress to someone (e.g., harming a person) is partially the result of experiencing empathic concern for the victim’s pain (Patil et al., 2017a) and partially stems from performing the bad action itself, resulting from aversive conditioning associated with the sensorimotor and perceptual properties of the harmful action (Cushman et al., 2012; Miller et al., 2014).

Studies on patients, lesions studies and neuroimaging research on healthy individuals strongly support the link between Empathy and Morality (Decety and Cowell, 2014): clinical populations characterized by deficits in processing empathy and emotions, such as patient with lesions in the ventromedial prefrontal cortex (vmPFC) or with Frontotemporal dementia, are more prone to endorse harmful actions compared to healthy controls (Mendez et al., 2005; Koenigs et al., 2007). Consistently, neuroimaging studies showed that evaluating personal harmful actions is associated with both relatively greater activation in brain regions associated with emotion processing regions, such as the medial prefrontal cortex or the Anterior Insula (Greene et al., 2001, 2004). Finally, individuals more prone to endorse harming one person in order to save more have been found to score low on an empathic concern scale (Gleichgerrcht and Young, 2013).

Given the importance of empathy and of processing emotions for morality, impaired development of these functions is consequently reflected in altered moral judgments (Patil and Silani, 2014a,b; Cardinale and Marsh, 2015), as occurs in individuals lacking or with decreased ability in these domains.

### Influence of Impaired Empathy- Emotional Processing on Moral Decision-Making Ability

In order to understand some of the decision-making deficits deriving from the reduction of emotional processing-empathy toward others, we introduce two personality conditions characterized by such difficulties as a “model” to understand potential mechanisms through which violence affects the normal development of emotional processing abilities and how moral decision-making ability of such individuals is consequently affected. Note that the studies introduced above consider psychopathy and alexithymia as dimensional constructs, rather than qualitatively distinct categories of behavior, and should be considered as an extreme variant of normal personality (Levenson et al., 1995; Hare and Neumann, 2005; Karukivi and Saarijärvi, 2014).

#### The Case of Individuals With Psychopathic Personality

Psychopathy is a personality construct characterized by emotional dysfunction, callousness, reduced remorse and antisocial behaviors due to poor behavioral control (Hare, 1999).^1^ Several studies have demonstrated that exposure to violence can result in increased likelihood of developing both the *antisocial* and the *interpersonal/affective* features of psychopathy later in life (Graham et al., 2012; Dargis et al., 2015), as coping mechanisms to avoid direct victimization (Dargis and Koenigs, 2017).

Importantly, these individuals are frequently profoundly impaired in both their ability to process affective information and to experience fear and guilt (Davidson et al., 2000; Herpertz and Sass, 2000). As a result, these individuals do not display the typical empathic response to distress of others (Blair et al., 2001; Blair, 2006). Previous research proposed that this blunted empathic response and reduced arousal toward others’ distress is responsible of the propensity for aggressive behavior in these individuals (Blair, 2007a). These emotional deficiencies are thought to be the result of neural dysfunctions in structures associated with fear-based learning and decision-making, such as the amygdala and the orbitofrontal cortex (Blair et al., 2006; Blair, 2007b, 2008): Meffert et al. (2013) found that brain regions involved in experiencing the affective components of social interactions (such as love, pain, and social exclusion) were not spontaneously activated as strongly in psychopaths, compared to healthy controls, while viewing video-clips displaying such emotions.

Research on *moral decision-making* in individuals with psychopathic traits of personality has yielded mixed results: some studies reported that this condition does not influence moral decisions (Tassy et al., 2009; Cima et al., 2010), whereas others report that this trait is associated with a greater rate of endorsing actions that condone harming fewer in order to promote greater welfare of many (Glenn et al., 2009; Bartels and Pizarro, 2011; Koenigs et al., 2012). Cardinale and Marsh (2015) explained these contrasting results distinguishing between judgments “about causing physical harm” and judgments “about causing emotional distress” and suggesting that psychopaths fail judgments of the latter category (specifically fear), but are able to correctly evaluate physical harm. The difficulties of individual with psychopathic traits to process others’ emotions have been hypothesized to be a long-term consequence of a lack of subjective experience during threatening situations, as demonstrated by reduced electrodermal activity and potentiated startle reflex during pain anticipation (e.g., Birbaumer et al., 2005; Blair et al., 2005; Marsh et al., 2011). By contrast they are able to judge causing physical harm to others to be morally unacceptable, because this kind of judgments is more based on semantic information about societal rules (Cardinale and Marsh, 2015).

Another study (Tassy et al., 2013) distinguished the ability to make *moral judgments* (“Is it appropriate to do X in order to”) which involves a “more abstract evaluation” of the situation with an allocentric perspective, from actual *endorsement of a behavior* (“Would you do X in order to”), which implies projecting oneself into a situation with an egocentric frame of reference, as responsible of differences in moral judgments found among psychopathic individuals (Frith and de Vignemont, 2005; Sood and Forehand, 2005). According to this perspective, higher levels of psychopathic traits do not predict differences in *judgments* during moral dilemmas, but they are associated with utilitarian responses in the case of *moral choices*: psychopaths are more likely to endorse a *choice decision* that would inflict suffering or death to an individual, if this lead to greater welfare of more people.

Neuroimaging studies (e.g., Glenn et al., 2009) clarified this pattern, showing that during moral decision-making higher psychopathy scores are associated with reduced activity in the vmPFC, a brain region involved in emotional processing and with increased activity in the right dorsolateral Prefrontal Cortex (dlPFC), involved in reasoning and cognitive control, thus suggesting that psychopaths may rely more on allocentric perspective of the situation when endorsing a decision.

Finally, Patil (2015) extended the understanding of moral dysfunction associated to psychopathic traits, investigating whether weaker negative affect experienced at the prospect of harming others is due to reduced harm aversion for harmful outcomes (*outcome aversion*) or for performing harmful actions (*action aversion*) (Cushman, 2013; Miller and Cushman, 2013; Miller et al., 2014). As mentioned, the aversion to actively causing distress to someone stems from both empathic concern for the victim’s pain, as well as from sensorimotor and perceptual properties associated with the harmful action (Cushman et al., 2012). In comparison to healthy controls, individuals with psychopathic traits have been found to display weaker discomfort from endorsing both harmful outcomes and actions and, consequently, they frequently endorse harmful actions and/or outcomes (Patil, 2015).

#### The Case of Individuals With Alexithymic Personality

According to the theoretical model of Taylor et al. (1991), Alexithymia is a dysfunctional personality construct, which could be present in different types of personality disorders or mental disorders, and which is defined by difficulties in identifying and describing one’s own feelings and a style of thinking relatively devoid of introspection.

This personality construct is of particular interest for the purpose of this paper as it has been associated with multifaceted difficulties in emotional processing (Taylor et al., 1991; Luminet et al., 2001, 2018) from recognizing emotions expressed by others (Parker et al., 1993; Prkachin et al., 2009; Swart et al., 2009; Grynberg et al., 2012), to regulating emotional responses (Swart et al., 2009; Pollatos and Gramann, 2012), and experiencing empathy toward others (Patil and Silani, 2014a,b; Cecchetto et al., 2017).

During typical development, children progressively learn to differentiate their own emotions from those of others’ through social interactions. However, exposure to aversive external circumstances, such as violence and/or caregivers not sufficiently empathic, can disrupt the ability to articulate and make sense of one’s own emotional experiences, leading children to feel emotionally overwhelmed (Craparo et al., 2014a,b; Edwards and Wupperman, 2017; Hébert et al., 2018). If this impairment is not quickly solved, it may involve a decreased emotional competence during growth (e.g., Lane and Schwartz, 1987; Taylor et al., 1997).

Individuals suffering alexithymic traits display compromised awareness of their own emotional states (Silani et al., 2008). This reduced capacity to recognize and attend to one’s own affective state in alexithymia is hypothesized to lead to impairments also in empathizing with others (for meta-analytic evidence, see Lamm et al., 2011). Indeed, high levels of alexithymia have been found associated with reduced activity in empathy-related neural circuits when alexithymic individuals are observing other people experiencing pain (Lockwood et al., 2013), confirming that difficulties in perceiving one’s affective feelings may blunt the perception others’ affective states (Moriguchi et al., 2007; Bird et al., 2010). Moreover, using the *Interpersonal Reactivity Index* measure of empathy (IRI – Davis, 1983), it was found that alexithymic individuals exhibit less empathic concern for others and reduced tendency for perspective-taking in both community and psychiatric/clinical populations (Guttman and Laporte, 2002; Silani et al., 2008; Grynberg et al., 2010; Patil and Silani, 2014a,b; for a review, see Bird and Cook, 2013).

It can readily be imagined that the reduced empathic concern alexithymic individuals have for a victim is reflected in their *moral judgments*. A clear example is offered in a study by FeldmanHall et al. (2012), in which participants were asked to make decisions about increasing their financial gain at the expense of another’s physical welfare (applying painful electric shocks). Higher levels of alexithymia traits were associated with delivery of higher levels of shock and more money kept, associated to a reduced activity in brain regions crucial for processing socio-emotional cognition, such as dorsal anterior cingulate cortex (dACC) and subgenual anterior cingulate cortex (sgACC). Furthermore, when individuals with alexithymic traits are presented with moral dilemmas asking if it is morally permissible to sacrifice the life of one to save more (the footbridge dilemma, Thomson, 1986), they are more likely to endorse utilitarian harmful actions (Patil and Silani, 2014b). Lastly, higher levels of alexithymic traits are associated with a reduced physiological activation during moral decisions (Cecchetto et al., 2017), during intentionality judgments (Zucchelli et al., 2019) and with atypical moral acceptability judgments: compared to controls, they consider less acceptable to endorse actions that result in happiness in others, and more acceptable to induce sadness, fear, disgust, and anger (Brewer et al., 2015). Importantly, recent research suggests that alexithymic traits are partly responsible of emotional processing difficulties reported by Autism Spectrum Disorder (ASD) patients when providing moral evaluations. In their study on ASD subjects, Patil et al. (2016) found that moral judgments of these individuals are influenced by both autistic traits, which were associated with reduced utilitarian bias due to a preserved emotional response to others’ affective states, as well as by alexithymic traits, which were instead associated with increased utilitarian bias due to their reduced empathic concern for the victim.

In summary, the psychopathic and alexithymic personalities examined here are useful to understand how an impaired development of empathy and emotional processing affects individuals’ moral decision-making abilities. According to the empathic blame hypothesis, the degree to which we condemn others for producing harmful outcomes, intentionally or unintentionally, closely depends on the degree to which we empathize with the victim’s suffering (Patil et al., 2017a). Without this perspective, our evaluation about negative effect of the outcome, and assessment of the agent’s responsibility for that act, will be altered. Relying on this evidence, one can therefore expect that individuals who display emotional deficits as a result of exposure to violence, will display similar moral behaviors to individuals with personality traits characterized by reduced emotional processing ability.

## Theory of Mind and Moral Decision-Making

### Exposure to Violence and Deficit in Theory of Mind

In this section, we focus our discussion on the ability to understand others’ mental states, i.e., Theory of Mind (ToM, Premack and Woodruff, 1978; Saxe and Kanwisher, 2003), another function which has been proposed to be necessary for the proper development and functioning of moral reasoning and behavior (Buon et al., 2016). Recent proposals have defined ToM as a multidimensional process comprising both cognitive and affective subcomponents. *Cognitive ToM* involves the ability to make inferences about beliefs and motivations of others, while *Affective ToM* relates to the ability of making inferences about the feelings of others (Baron-Cohen and Wheelwright, 2004; Shamay-Tsoory et al., 2010; Dvash and Shamay-Tsoory, 2014). Note that while, sometimes, affective ToM and cognitive empathy have been considered as similar, here we treat them as two different systems – following the theoretical model of Shamay-Tsoory et al. (2009) and Kalbe et al. (2010). This perspective holds that Affective ToM involves understanding the mental states- thoughts and emotions- of others (Frith and Frith, 1999), whereas cognitive empathy involves adopting another individual’s psychological perspective, “putting themselves in their shoes” (Dvash and Shamay-Tsoory, 2014).

Recent studies indicated that both components of ToM are impaired in individuals who experienced violent traumas. Nazarov et al. (2014) found that compared to healthy controls, women with post-traumatic stress disorder (PTSD), had difficulties in interpreting scenes depicting familiar interactions (competition, deception, and intimacy), where the information necessary for interpreting these interactions had to be deduced from non-verbal cues. Additionally, these individuals took longer than controls to perform tasks measuring recognition of complex mental states from emotionally salient facial/eye expressions. A more recent study (Germine et al., 2015) examined how ToM may be affected by a large amount of violent and non-violent traumas, including physical abuse, sexual abuse, neglect, and parent loss, as well as negative parental behaviors related to drug abuse, alcoholism, and criminal activities. Results showed that childhood experiences of parental maltreatment (primarily physical abuse) and parental maladjustment (e.g., parental criminality, alcoholism, and drug abuse) were strongly associated with reduced ability to accurately infer other people’s mental states. Moreover, Quidé et al. (2017) demonstrated that childhood exposure to trauma also leads to alterations in the functioning of brain regions necessary to the understanding of others’ mental states (including the temporal parietal junction -TPJ, posterior cingulate cortex-PCC, and dorsomedial prefrontal cortex – dmPFC), and with the likelihood of being diagnose with behavioral disorders such as Schizophrenia.

As for the emotional processes discussed in the previous section, *desensitization* has been proposed as one of the mechanisms that can explain how violence can lead to ToM deficiencies. *Cognitive desensitization* is a process that consists in a reduction in reactivity to a given stimulus following a prolonged exposure to it. In the case of violence, it is suggested that prolonged exposure leads an individual to believe that violence is mundane and inevitable, decreasing the likelihood that it will be censured or perceived negatively. In other words, initial exposure to violence may lead to a degree of cognitive aversion, but after repeated exposure, individuals may develop violence- supporting beliefs- a process referred to as “violence normalization” (Funk et al., 2003; McMahon et al., 2009). This mechanism involves a generalized delay in the development of Theory of Mind ability: being repeatedly exposed to some mental states, like anger, individuals become less able to recognize and understand different emotions, ending to identify signs of anger in other people even if not present (Pollak et al., 2000).

In the following paragraph, we discuss how Theory of Mind serves moral decision-making ability.

### Theory of Mind and Moral Decision-Making

Theory of Mind is another of the crucial processes necessary for moral decision-making development and proper functioning. Several theoretical proposals have characterized how Theory of Mind may be involved in morality (e.g., Knobe, 2005; Young et al., 2007). In particular, Cushman’s (2008) dual-process model of morality suggested the existence of two different systems, one involving the evaluation of the agent’s causal responsibility (whether an agent caused harm) and the other involving the evaluation of the agent’s mental state (the agent’s intention for the outcome) as necessary to elaborate moral evaluations. Crucially, this latter element fundamentally relies on ToM abilities to produce moral judgment about agents. Indeed, understanding others’ mental states is necessary to understand the intention of an agent and, consequently, in order to be able to attribute blame and praise to him: an agent who intentionally committed harm will be blamed more than one who caused harm accidentally, vice-versa someone who helped others intentionally will be praised more than someone who did so accidentally (Lagnado and Shannon, 2008).

Neuroimaging, developmental research, and studies on patients provide a decisive support to the relationship between Theory of Mind and moral decision-making ability. Neuroimaging studies indicated that: (i) when making moral judgments about accidental harms (unintended harms) people express more lenient condemnations of the harmful action because unwanted, and that such behavioral effect was mirrored in the brain by an increase in activation of the right TPJ, one of the critical neural nodes associated with ToM (Koster-Hale et al., 2013); (ii) right TPJ is also involved when making judgments on attempted harms (e.g., actors who intended but failed to do harm so characterized by a negative intent but a neutral outcome), as in this case expressing a moral judgment requires taking into account the beliefs of the agent (Young et al., 2007). Importantly, Young et al. (2010), established a causal role for this brain area to integrate ToM information in moral judgments, demonstrating that individuals with reduced activity in the right TPJ express more lenient moral judgments in the case of attempted harms; (iii) local gray matter volume in the left anterior superior temporal sulcus (STS), also part of the mentalizing network, was associated with the degree to which participants relied on information about innocent intentions to forgive accidental harm in moral judgments (Patil et al., 2017b).

Consistently, developmental studies showed that, when presented with moral scenarios characterized by a conflict between agent’s intentions and the outcome of an action, younger children’s moral judgments are mainly based on outcomes rather than on intentions (e.g., Zelazo et al., 1996). As children develop more mature TOM capacities, however, they become progressively able to integrate belief information in moral judgment, understanding the accidental nature of a person’s actions (Fu et al., 2014).

Finally, patients with ASD and individuals with autistic trait of personality, characterized by reduced ToM ability (American Psychiatric Association, 2013; Gökçen et al., 2014, 2016) have been found to produce moral and intentionality judgments mostly relying on outcomes of actions rather than on the agent’s intention (Fadda et al., 2016; Zucchelli et al., 2018).

Jointly, these studies support the view that moral judgments rely on a sophisticated understanding of others’ mental states and on the ability to differentiate intentional and unintentional actions (Loureiro and Souza, 2013).

### Influence of Impaired Theory of Mind on Moral Decision-Making Ability

In this section, we introduce two personality disorders, both characterized by deficits in Theory of Mind, as a “model” to understand how deficiencies in this ability affect moral decision-making. Note that, in this paragraph, we focused on studies testing individuals diagnosed with a clinical disorder.

#### The Case of Borderline Personality Disorder

According to the DSM5 (American Psychiatric Association, 2013) Borderline Personality Disorder (BDL) is a condition characterized by a variety of deficits including mood instability, loss of sense of self, and – most importantly for the purposes of this paper – a deficit in the mentalizing ability (Sharp et al., 2011). In particular, these individuals appear to be impaired in complex ToM tasks, such as the “Movie for the Assessment for Social Cognition”—MASC test (Dziobek et al., 2006), but not on simpler ToM tasks (such as the “Reading the mind in the eyes” test, Baron-Cohen et al., 2001). Interestingly, the MASC test allows to characterize ToM deficits based on incorrect responses: over-mentalizing errors (excessive attribution of mental states), reduced ToM (defective attribution of mental states), and lack of ToM (lack of mental state concept). Using MASC, Sharp et al. (2011) and Vaskinn et al. (2015) found that both BDL adolescent and adult individuals were characterized mainly by impaired ToM due to overmentalizing errors. Since greater ToM dysfunctions in BDL have been often found to occur in association with post-traumatic stress disorder or a history of sexual trauma (Zanarini et al., 2002; Preißler et al., 2010), it has been suggested that the tendency to overmentalize relates to a defensive mechanisms elicited by these traumatic experiences (Gibb et al., 2001; Rogosch and Cicchetti, 2005; Sieswerda et al., 2007; Vaskinn et al., 2015).

Crucially, the deficit in the ability of perspective taking in BDL patients is reflected in their impaired *moral decision-making*. In particular, BDL individuals lack a feeling of personal identity and consequently have difficulties in perceiving self and other as separate individuals (Minkowitz, 1961). This has large impacts on moral attribution: for instance inmates diagnosed with BDL personality exhibit difficulties in assigning blame to others (Wolf et al., 1995). Indeed, when required to consider the perspective of several other agents in order to assign blame in a series of vignettes describing crimes, BDL subjects were able to make judgments by focusing on the perspective of only one agent, reflecting a difficulty in taking into consideration multiple points of view (Wolf et al., 1995).

#### The Case of Schizophrenic Disorder

The second personality pattern introduced, characterized by defective Theory of Mind, is Schizophrenia. According to the DSM5 (American Psychiatric Association, 2013) Schizophrenia is characterized by two or more of the following symptoms appearing for at least 1-month (or longer): delusions, hallucinations, disorganized speech, disorganized or catatonic behavior, diminished emotional expression and impairments in one of major areas of functioning like work, interpersonal relations, or self-care. This condition has been often found related with exposure to violence like physical abuse, sexual abuse or witnessing domestic violence (Rosenberg et al., 2007; Vallejos et al., 2017).

Several studies have found associations between Schizophrenia and reduced ToM ability (Shamay-Tsoory et al., 2007), the complete disregard of others’ mental states (Montag et al., 2011) or, at the opposite extreme, an excessive interpretation of others’ beliefs (Frith, 1992; Abu-Akel and Bailey, 2000). Vaskinn et al. (2015) analyzed these difficulties through the MASC test, finding that these individuals exhibit both under-mentalizing and over-mentalizing errors (excessive attribution of mental states), and that these latter were also associated with high rate of hallucinations and delusions (drawing conclusions and inferences that were not based on existing evidence). Finally, a meta-analysis of 29 studies, with a total sample size of over 1500 participants, indicated that, on average, the Theory of mind performance of participants with schizophrenia is more than one standard deviation below that of healthy controls (Sprong et al., 2007).

The investigation of *moral decision-making* in schizophrenia has started in the last century, with Johnson (1960) suggesting that people with schizophrenia treat others in a way “hardly different from inanimate objects and they reject responsibility for the welfare of others” (p. 283).” Further studies on moral cognition and schizophrenia used Kohlberg’s Moral Judgment Interview (MJI; Colby et al., 1987), a semi-structured interview which investigates moral judgments in the context of several moral dilemmas, including the so-called “Heinz dilemma” which describes a man who considers stealing an expensive drug to save his wife’s life. Participants were asked whether Heinz should steal the drug, followed by a series of probes investigating the relevance of duty, love, property rights and right to life for the scenario. People with schizophrenia obtained lower scores, focusing their explanations on “power, status and possessions, compared to concepts of equality, reciprocity and trust” found in control participants (Benson, 1980, p. 676). According to McGuire et al. (2014) this worst performance was linked to the inability of these individuals to correctly make inferences, required from the MJI, about the mental states of the agents involved. Interestingly, Langdon et al. (2014) examined 43 patients with schizophrenia and healthy controls on Theory of mind tests and on a novel test of social judgment (adapted from autism research – Ellis et al., 1994). This latter measure required participants to judge whether various social behaviors were normal or reasonable in the context in which the behaviors occurred. Specifically, participants were asked to judge three types of behavior, as either normal, unusual, or shocking: (1) behavior that is appropriate according to social norms; (2) violations of social norms; and (3) impolite behavior that is understandable if the agents’ thoughts are taken into account. Results showed that while patients had clear deficits in ToM, they performed similarly to controls when judging socially appropriate behaviors and violations of social norms. Importantly, however, individuals with schizophrenia were less likely than controls to judge social behavior as reasonable when the behavior was, while impolite, justifiable if the agents’ thoughts were taken into account. Thus, these findings confirm that ToM deficits impair the ability of schizophrenic individuals to consider other people’s thoughts and intentions when making evaluations of social behaviors, resulting in atypical moral judgments.

Overall, the disorders examined in this section illustrate how reduced development of ToM affects moral decision-making. In particular, deficits in ToM lead to disregard toward others’ mental states and/or misinterpretations of others’ beliefs, and a consequential increase in self-reference, that ultimately result in a tendency of moral decisions grounded mostly on the outcomes of actions, ignoring the intentions moving such actions. Lack of ToM is proposed to be particularly severe in the case of attempted harms, in which the lack of negative outcomes can lead patients to ignore the negative intentions driving a behavior, leading to abnormal moral judgments. For this reason studies exploring moral judgments about attempted harms in individuals with Borderline Personality disorder and Schizophrenia disorder, would be useful to investigate more accurately the moral decision making of these populations.

## Inhibitory Control and Moral Decision-Making

### Exposure to Violence and Deficit in Inhibitory Control Ability

Inhibitory control is the ability of an individual to inhibit impulses and habitual or dominant behavioral responses to stimuli, in order to select the more appropriate behavior in the contingent situation. Recent studies are bringing increasing attention to the impact of chronic violence on the development of inhibitory control abilities, in particular given their importance for proper moral development (Buon et al., 2016). Bogliacino et al. (2017) analyzed the effects of prolonged exposure to violence on short-term memory and cognitive control, in a sample of Colombian civilians who were exposed either to urban violence or to warfare more than a decade earlier. They asked to one group of participants to recall experiences of violence, compared to another who was requested to recall emotionally neutral or joyful experiences, before the execution of cognitive tests measuring working memory and cognitive control abilities. Results indicated that exposure to violence significantly reduces both short-term memory and cognitive control, but only in the group that was actively recalling emotional states of fear and anxiety linked with experiences of violence. On the contrary, when such emotional states were not recalled, accuracy on memory and cognitive tasks was no different between groups. These findings suggest that unresolved traumatic experiences, that are frequently remembered, may significantly negatively affect a person’s ability to exercise short-term memory and cognitive control abilities. This dysfunction thus affects a person’s ability to function well in life, even after more than a decade had passed from the main traumatic event that was recalled. Bogliacino et al. (2017) characterized the following mechanisms through which violence leads to inhibitory control dysfunctions: violence repeatedly triggers automatic reactions in our decision-making systems, devoted to developing strategies capable of managing a threatening situation. In the long run, being constantly stimulated, this impulsive responses to stimuli become the predominant way in which a person responds to unconditioned stimuli, instead of relying also on other decision systems, such as the more reflective ones. Additional support for this hypothesis comes also from neurobiological studies, which showed that acute stressors impact the neuroendocrine system underlying cognitive control. Specifically, stressors lead to a high release of catecholamines, which impair cognitive control related activity in the prefrontal cortex (PFC), and at the same time enhance the habitual responses of the amygdala and of the dopamine system (Phelps and LeDoux, 2005; Arnsten et al., 2015). The defective amygdala– pregenual anterior cingulate cortex (pgACC) regulatory circuitry then results in a heightened sensitivity to conflicting emotional information and a lack of regulatory control over emotion processing (Marusak et al., 2015). The negative effect of violence on inhibitory control ability was also demonstrated by other studies looking at patients diagnosed with PTSD: when performing a continuous performance tasks (CPT), a task which measures the ability to maintain a consistent focus on some continuous activity while ignoring competing stimuli, these individuals made more errors of commission, due to distracter stimuli, compared to healthy controls (Vasterling et al., 1998). Increased errors among PTSD patients were further related to both the severity of re-experiencing the traumatic events, as well as to connected hyperarousal symptoms (Vasterling et al., 1998).

In sum, early life exposure to violence has been associated with reorganization of neural circuits in ways that enhance automatic and impulsive processing of salient emotional stimuli as a defensive strategy, at the expense of a decreased development of the reflective and control systems.

### Inhibitory Control Ability and Moral Decision-Making

As discussed in the previous sections, recent models in moral psychology suggest that moral judgment and behavior rely on the interaction of multiple decision systems. These evaluative systems enable individuals to decide what is morally right or wrong (Cushman et al., 2007; Cushman, 2008; Greene, 2009). The ETIC model of morality (E = emotional arousal, T = theory of mind, IC = inhibitory control – Buon et al., 2016) offers a detailed characterization of how emotional responses, ToM competencies and inhibitory control abilities interact in order to produce moral evaluations. This model proposes that emotional arousal, elicited by observing someone else’s distress (Seara-Cardoso et al., 2012), generates an automatic and negative reaction to a victim’s distress. ToM capacities, instead, figure out an agent’s intentions and, finally, inhibitory control resources are required to resolve conflicts if the outputs from the two previous systems are incongruent. One example of situations where these two systems yield contrasting information are cases of *accidental harms*: a negative emotional assessment is elicited by the distress of the victim, however, a neutral evaluation of the agent’s action follows from his blameless intentions. In such cases, then, inhibitory control suppresses the negative emotional arousal elicited by the victim’s suffering, enabling the agent’s neutral intention to determine the final moral evaluation. The role of inhibitory control abilities in moral judgment is also supported by a recent fMRI study (Treadway et al., 2014), which showed that when participants judge accidental harm, the dorsal anterior cingulate cortex (dACC), one of the brain regions typically associated with conflict monitoring and cognitive control (Botvinick et al., 2001), exhibits enhanced functional connectivity with the amygdala. From a development perspective, a significant development of inhibitory control abilities occurs only during adolescence (Best and Miller, 2010). This is consistent with observations that when making moral judgments of agents causing harms to other, younger children tend to rely just on the discomfort conveyed by their emotional systems which are already fully developed, ignoring information related to intentions, which have been suggested to develop only later in life (Fu et al., 2014). Afterward, with the development of ToM capacities, children become able to produce intention-based moral evaluations, but only when there is no conflict between intentions and outcomes. The ability to down-regulate the discomfort produced by accidental harm, allowing to focus on the neutral intentions of the agent, has been proposed to emerge only later along with the development of inhibitory control resources (Buon et al., 2016).

### Influence of Impaired Inhibitory Control on Moral Decision-Making Ability

Given the importance of inhibitory control for morality, it is plausible to expect that when this ability is defective, individuals will also display impaired moral behavior and judgments. Hereafter we discuss evidence illustrating how morality decision-making of individuals diagnosed with reduced inhibitory control differs from those of healthy controls. Note that the studies discussed hereafter were all done on patients who were clinically diagnosed with the respective disorder.

#### The Case of Antisocial Personality Disorder

According to DSM5 (American Psychiatric Association, 2013) Antisocial Personality is diagnosed in individuals who persistently disregard for moral codes and social norms since age 15, frequently being arrested for violating laws, and display impulsive behaviors such as high irritability and aggressiveness. An increased likelihood of developing this condition has been found associated with having antisocial parents, being male, living in low socio-economic status households, belonging to a minority group (Farrington, 2006) and being exposed to greater levels of violence. This last element has been proposed to increase the development of this disorder, as it provides examples for deviant behaviors, which increase the perceived acceptability of antisocial behavior and ultimately desensitize the emotional inhibitory effects of violent immoral behavior (Guerra et al., 2003; Mrug and Windle, 2009; Voisin et al., 2016; Kersten et al., 2017).

Antisocial Personality Disorder (APD) has been consistently associated with cognitive control and inhibitory deficits (Morgan and Lilienfeld, 2000). For instance, Verona et al. (2012) examined these difficulties by recording event-related brain potentials during an emotional-linguistic *Go/No-Go* task to examine modulation of negative emotional processing by inhibitory control. Healthy participants suppressed negative emotional processing under conditions requiring inhibitory control, and their frontal P3 increases as a function of inhibitory control (No-Go P3; Smith et al., 2008); on the other hand the APD group was unable to inhibit negative emotional processing, focusing more on negative emotional versus neutral words regardless the cognitive control demands (both *Go* and *No-Go* conditions). The authors concluded that these individuals are unable to ignore the processing of negative emotion, even when this processing is maladaptive.

Several studies examined *moral decision-making* in patients with APD (for reviews see Blasi, 1980). More in detail, Stams et al. (2006) conducted a meta-analysis of 50 studies to investigate whether, compared to non-delinquents, juvenile delinquents were found to have lower levels of development in moral reasoning, defined in accordance with Kohlberg’s (1973) moral development model. Briefly, Kohlberg’s model proposes that moral development can be characterized by six hierarchically ordered stages, consisting of three different levels of moral reasoning: preconventional, conventional, and postconventional. Achieving each of these levels requires a development in reasoning abilities, that are typically unfolding as a person develops (Colby et al., 1987). In particular, reaching each stage requires a development in the capacity to provide more profound and universally acceptable solutions to moral issues, whereas earlier stages are typically characterized by pronounced egocentric biases, where moral judgments are mainly focused on one owns feelings and intuitions. The results of this meta-analysis revealed that the development of moral reasoning in juvenile delinquents is substantially lower compared to that of non-delinquents. Briefly, Kohlberg’s model proposes that moral development can be characterized by three different levels of moral reasoning preconventional, conventional, and postconventional. Furthermore it showed that the effect of being incarcerated on normal moral reasoning development was moderated by some factors like gender (male offenders had lower scores of moral development) and IQ level (people with lower IQ had lower moral development). In short, this meta-analysis revealed that moral reasoning in patients with APD, a condition associated with lower inhibitory control abilities, is significantly less developed than in healthy controls.

#### The Case of ADHD

According to DSM5 (American Psychiatric Association, 2013) ADHD condition is characterized by a persistent pattern of inattention and/or hyperactivity-impulsivity that interferes with functioning or development. Individuals with ADHD present deficits in response inhibition, planning, and cognitive flexibility when compared to control individuals (Willcutt et al., 2005; Kuntsi et al., 2014). Although genetic heritability is linked to the etiology of the disorder (Brassett-Harknett and Butler, 2007) adverse childhood events (Biederman, 2005), early traumatic events (Briggs-Gowan et al., 2010), community violence (Fishbein et al., 2009) and child abuse (Briscoe-Smith and Hinshaw, 2006) have been also linked to increased likelihood of developing ADHD (Lewis et al., 2015).

Even though the impact of ADHD on *moral decision-making* is still not fully characterized, a number of studies suggest that both children and adolescents with ADHD may be at risk for less mature moral development due to their inhibitory control deficits (Nucci and Herman, 1982; Barkley, 1998; Buon et al., 2016). One previous study (Chung et al., 2013) tested if patients with ADHD treat moral norms differently compared to healthy controls. Here it was shown that ADHD patients show a relatively heightened tendency to make moral judgments on the basis of cost-benefit analysis rather than abstract-idealistic moral rules (e.g., do not harm others). Based on the evidence proposed in Chung et al. (2013), one can expect that in sacrificial moral dilemmas (such as that footbridge dilemma) these individuals will focus more on the anticipated consequences of actions than on moral principles. Similarly, in scenarios that require the evaluation of both the intentions and outcomes of a given action (e.g., cases of attempted or accidental harm), one could expect that ADHD patients’ moral judgment will rely mostly on the outcomes of the action rather than on the intentions. Therefore, future research is needed to determine the scope and characterization of these expected deficits.

In summary, some evidence suggests that both individuals with Antisocial Personality disorder and ADHD disorder have less developed moral reasoning, but the origins of these dysfunctions are still understudied. Future studies investigating moral judgments about accidental harms in these individuals could characterize better how these disorders impact decision processes involved in moral judgments. Thus the investigation of such hypotheses is currently starting.

## A Vicious Cycle: Impaired Moral Judgments and Increased Violence

So far, we have discussed how violence causes deficits in emotional processing-empathy, Theory of mind, and inhibitory control mechanisms, that result in abnormal moral decision-making (see Table 1). In this section we discuss evidence showing that deficits in these decision processes, in turn, directly promote violent behavior. In other words, this section highlights how impaired moral decision-making abilities can ultimately resulting in a vicious self-reinforcing cycle of violence (Spenser et al., 2015).

**TABLE 1:** Summary of deficits in moral decision-making deriving from disruption of Empathy, Theory of Mind and Inhibitory abilities following exposure to violence.

***Ability***	***Function***	***Personality conditions***	***Deficit in moral decision-making***
Empathy	*Necessary to appreciate the distress of the victim (Buon et al., 2016; Patil et al., 2017a)*	**Psychopathic traits of personality** (Blair et al., 2001; Blair, 2006)	Cardinale and Marsh (2015): psychopathic individuals fail moral judgments about causing emotional distress in others.
			Tassy et al. (2013): psychopathy traits are associated with utilitarian responses in the case of moral choices.
			Patil (2015): psychopathy individuals display weaker discomfort from endorsing both harmful outcomes and action in moral judgments.

		**Alexithymic traits of personality** (Silani et al., 2008; for meta-analytic evidence, see Lamm et al., 2011)	Patil and Silani (2014b): alexithymic individuals showed higher endorsement of utilitarian solution in moral judgments.
			Gleichgerrcht and Young (2013): some alexithymic individuals showed to be intransigent utilitarians (endorsing utilitarian solution for both impersonal and personal moral dilemmas).

Theory of mind	*Necessary to appreciate the intentions of the agent, particularly in the case of attempted harms (Young et al., 2007; Buon et al., 2016)*	**Schizophrenic disorder** (Blair et al., 2001; Blair, 2006)	McGuire et al. (2014): schizophrenic individuals exhibit lower moral maturity scores on the Kohlberg’s Moral Judgement Interview because of their inability to correctly make inferences about the mental states of the agents involved in the moral dilemmas.
			Langdon et al. (2014): individuals with schizophrenia were less likely to judge social behaviour as reasonable when the behaviour was, while impolite, justifiable if the characters’ thoughts were taken into account.

		**Borderline Personality Disorder** (Sharp et al., 2011; Vaskinn et al., 2015)	Wolf et al. (1995): when required to consider several agents’ perspective in order to assign blame in moral judgments, individuals with Borderline disorder were able to make judgments by focusing on the perspective of only one agent, reflecting a difficulty in taking into consideration multiple points of view.

Inibitory control ability	*Necessary to down regulate the emotional reaction to victim’s distress and to consider agent’s intention, particularly in the case of accidental harms (Buon et al., 2016)*	**Antisocial personality disorder ** (Guerra et al., 2003; Pearce et al., 2003; Cecil et al., 2014; Voisin et al., 2016)	Stams et al. (2006): development of moral reasoning in juvenile delinquents is substantially lower compared to that of non-delinquents.

		**ADHD disorder** (Kuntsi et al., 2014)	Chung et al. (2013): individuals with ADHD show a relatively heightened tendency to make moral judgments on the basis of cost-benefit analysis rather than on abstract-idealistic moral rules (e.g., do not harm others).

Several studies (Palmer, 2003, 2005; Bzdok et al., 2012; Spenser et al., 2015) have recently suggested that when moral reasoning in an individual only develops to a limited extent, i.e., to immature levels, this person’s moral judgments will be affected by a series of cognitive distortions, that frequently lead to considering morally appropriate to behave in antisocial ways, such as harming others. Specifically, an immature moral reasoning, due to defective ability to understand the believes and intentions of others, their emotional states – especially those defined as moral emotions such guilt, shame or empathy – and to control one’s own emotional reactions has been linked to a series of cognitive distortions (Gibbs, 1993) such as: (a) “egocentric bias,” i.e., the tendency to rely too heavily on one’s own perspective, with inability to take other people’s perspective and to inhibit emphasis on one’s own needs ahead of those of other people; (b) “shifting of responsibility” which involves attribution of blame and intent onto others when hurting someone; (c) “minimization/mislabeling” of wrongful behavior and its consequences; and (d) “misinterpretation of others” actions as intentional rather than accidental’ (Dill et al., 1997). These biases are common features among offenders, particularly in order to justify their crimes and minimize feelings of guilt or regret they find hard to otherwise explain, and by doing this, inhibition to aggression and harmful behaviors is undermined (Palmer, 2000).

Importantly, these cognitive distortions jointly make it more likely that ambiguous social cues will be interpreted through the lenses of a hostile attribution bias, which, in turn, triggers negative beliefs about social interactions. Indeed, individuals exhibiting these biases have been found to pay less attention to social cues prior to interpreting a situation (Dodge and Newman, 1981), to rely more on internal schema to interpret situations (Dodge and Tomlin, 1987) and to selectively attend to aggressive cues (Gouze, 1987).

Moreover, these negative beliefs can then be used as a filter to understand future experience, leading to an increase of aggressive and antisocial actions toward others, particularly in situations emotionally arousing, as a defensive response (Palmer, 2000).

Multiple studies have confirmed that the risk of violence is higher among individuals lacking empathy, Theory of mind and inhibitory control abilities and, in turn, a defective moral reasoning ability.

Comprehensive reviews (Fazel et al., 2009; Nielssen and Large, 2010) found an increased risk of violent behavior in individuals with *schizophrenic disorder* (*N* > 18.000), in particular with respect to extreme violence (e.g., homicides). More specifically, these research found that people with schizophrenia comprise between 5 and 20% of all homicide offenders, and that 12% of people with schizophrenia self-report a history of assaultive behavior.

Similarly, numerous studies have identified *psychopathic personality* as a reliable predictor of violence across varied populations (for a review see Hare, 2003): individuals with a psychopathic personality have been found to be more likely, than non-psychopathic individuals, to behave aggressively, using instrumental aggression or to be impulsive and, in turn, being convicted (Serin, 1991). Moreover, Walsh et al. (2007) examined how different aspects of the psychopathic personality may be differentially related to specific types of violent behavior, using the Hare’s Psychopathy Checklist (Hare, 2003), which investigates *interpersonal/affective* as well as *antisocial conduct/style of life* features of psychopathy. This research found that scores on the *affective subcomponent* of psychopathy were positively associated with domestic violence, whereas both *interpersonal* and *antisocial subcomponents* of psychopathy were positively associated with instrumental aggression.

Affect dysregulation and related problems with impulsivity, anger control and aggression constitute a characterizing symptomatic pattern also in *Borderline Personality Disorder*. Aggression in BDL patients manifests itself in self-destructive behavior (e.g., high risk behavior, self-injury) or externally directed aggression (Látalová and Prasko, 2010). Previous studies, which used self-rating scales (i.e., the State-Trait Anger Expression Inventory- STAXI- Spielberger and Sydeman, 1994 and the Buss-Perry Aggression Questionnaire – BPAQ, Buss and Perry, 1992), as well as behavioral assessments of aggression (the Point Subtraction Aggression Paradigm- PSAP – Cherek, 1992) revealed elevated levels of anger and aggression in BDL patients (Dougherty et al., 1999).

Importantly, people’s difficulty in understanding and expressing emotions, like in *Alexithymic personality*, may also increase the likelihood of responding to a situation with violence (Strickland et al., 2016; Velotti et al., 2016). These studies indicate that deficits in understanding and tolerating emotions are associated with defective emotion regulation strategies, that may lead to use aggression in order to terminate negative affective states. In line with this, Strickland (2014) examined violent offenders incarcerated and men from the general community, finding that the first group scored significantly higher on total alexithymia score, in particular with respect to the subscale of the Toronto Alexithymia Scale (TAS-20, Taylor et al., 2003) that measures difficulty in identifying and describing feelings. This confirms that their difficulties are related to the management of emotional situations.

Finally, over the past few years, there has been a growing interest in the association between *ADHD* and violence. Cross-cultural studies reported high prevalence rates of ADHD in male prisoners, with rates of up to 71% (for review see Young and Thome, 2011). Alarmingly, there is a strong association between ADHD and episodes of reactive aggression among these individuals (Retz et al., 2004).

Taken together, this body of research indicates that individuals diagnosed with Psychopathic and Alexithymic personalities, Schizophrenic, Borderline, ADHD and Antisocial personality disorders, each characterized by difficulties in either empathize with others’ emotions, understanding others’ mental states, or exert inhibitory control, are not only the result of exposure to violence, but also the precursors of violent behavior.

## Conclusion

The present review suggests that exposure to violence substantially increases the dysfunction of mechanisms necessary for proper moral decision-making ability, in particular Empathy, Theory of Mind and Inhibitory control. We relied on studies examining a series of disorders and personality traits, characterized by deficiencies in each of these abilities, to propose a highly plausible “model” of how exposure to violence can disrupt moral reasoning as a result of impairing each of those abilities. As morality is necessary in order to promote cooperation and limit aggressive interactions across individuals (Rai and Fiske, 2011; Greene, 2015), its disruption is frequently associated with increased risk of violence. A better understanding of the underling mechanisms, explaining how exposure to violence leads to impaired moral behavior is required in order to provide a solid scientific basis for interventions aimed at breaking this negative relation. To this end, our review proposes that strategies dedicated at mitigating the negative effects of violence have great chances of succeeding by targeting psychological mechanisms related to Empathy, Theory of Mind and Inhibitory control abilities. Encouragingly, recent prosocial behavior interventions have shown effective results in improving these abilities and could be used to disrupt the negative effects of violence on these mechanisms and, consequently, to ameliorate moral decisions among individuals displaying the negative behavioral effects resulted from having been exposed to violence (Chiu Ming Lam et al., 2011; Gvozdic et al., 2016; Mesurado et al., 2018).

## Author Contributions

Both authors listed have made a substantial, direct and intellectual contribution to the work, and approved it for publication.

## Conflict of Interest Statement

The authors declare that the research was conducted in the absence of any commercial or financial relationships that could be construed as a potential conflict of interest.

## Footnotes

1 Importantly, beside sharing some features with the Antisocial Personality Disorder (APD), such as impulsivity, irresponsibility, and failure to conform to social norms, Psychopathic personality is distinguished from APD by the presence of Fearless-Dominance and Boldness as well as a severe emotional dysfunction especially in experiencing empathy for others (e.g., Harpur et al., 1988; Hare et al., 1990; Decuyper et al., 2009; Anton et al., 2012; Hyde et al., 2014). Therefore, in the present paper, the two disorders will be considered separately, in line with several previous works of Hare et al. (1991), Sarkar et al. (2011), Wall et al. (2015), and will be discussed in relation to different dysfunctions (processing of emotions-empathy deficit the former vs. inhibitory dysfunction the latter). Note also that we do not adopt the Psychopathy definition of Diagnostic and Statistical Manual of mental disorders- DSM III/DSM5 (American Psychiatric Association, 2013), as it is unclear whether the DSM5 description of psychopathy is sufficiently accurate, as it is only focused on the antisocial features of this disorder but does not consider the more primary characteristics of psychopathy, namely Fearless-Dominance and Boldness (Lilienfeld, 1994).
